# The Impact of Arthroscopic Capsular Release in Patients with Primary Frozen Shoulder on Shoulder Muscular Strength

**DOI:** 10.1155/2014/834283

**Published:** 2014-06-24

**Authors:** Michał Waszczykowski, Michał Polguj, Jarosław Fabiś

**Affiliations:** ^1^Department of Arthroscopy, Minimally Invasive Surgery and Sports Traumatology, Medical University of Lodz, 113 Żeromskiego Street, 90-549 Lodz, Poland; ^2^Department of Angiology, Chair of Anatomy, Medical University of Lodz, 60 Narutowicza Street, 90-136 Lodz, Poland

## Abstract

The aim of this study was to evaluate the impact of arthroscopic capsular release in patients with primary frozen shoulder on muscular strength of nonaffected and treated shoulder after at least two-year follow-up after the surgery. The assessment included twenty-seven patients, who underwent arthroscopic capsular release due to persistent limitation of range of passive and active motion, shoulder pain, and limited function of upper limb despite 6-month conservative treatment. All the patients underwent arthroscopic superior, anteroinferior, and posterior capsular release. After at least two-year follow-up, measurement of muscular strength of abductors, flexors, and external and internal rotators of the operated and nonaffected shoulder, as well as determination of range of motion (ROM) and function (ASES) in the operated and nonaffected shoulder, was performed. Measurement of muscular strength in the patient group did not reveal statistically significant differences between operated and nonaffected shoulder. The arthroscopic capsular release does not have significant impact on the decrease in the muscular strength of the operated shoulder.

## 1. Introduction

Frozen shoulder is an inflammatory condition of the shoulder joint area manifesting with pain and limitation of range of passive and active motion in this joint. Primary frozen shoulder, involving limitation of the joint mobility without any apparent cause, is the most common form of the condition. Frozen shoulder may also occur after a trauma (posttraumatic frozen shoulder) or surgery in the shoulder area or be concomitant with diabetes mellitus, rheumatoid arthritis, or thyroid diseases [1–6].

Management of choice involves conservative treatment. Nonsteroidal anti-inflammatory drugs, oral or intra-articular glucocorticosteroids, physical therapy, kinesitherapy, and forced manipulation under general anaesthesia are applied [3, 5, 7–12]. In some 10% of cases indications for surgery are present. Currently, arthroscopic capsular release is a treatment of choice in such cases [3, 4, 13–19]. However, there are no literature data concerning the impact of such treatment on the muscular strength of the operated shoulder. Many data show the improvement of function of the shoulder after surgical treatment. But we still do not know whether it depends only on improvement of pain-free range of motion or it also correlates with good muscular strength of operated shoulder. We suppose that improvement of function of the shoulder after arthroscopic capsular release depends on good pain-free range of motion as well as good muscular strength of the shoulder. The purpose of this study is to determine whether good function of the shoulder after at least two-year follow-up after surgical treatment of frozen shoulder correlates with good muscular strength.

## 2. Materials and Methods

The analysis involved 27 patients treated surgically in the years 2006–2010 in the Department of Arthroscopy and Sport Traumatology, Medical University of Lodz, due to primary frozen shoulder with at least two-year follow-up. The group of 27 patients (27 shoulders) comprised 17 females and 10 males. Mean age in the whole group was 51.6 years ± 11.5 (24–76).

The inclusion criteria for this study were as follows: first episode of frozen shoulder, at least 6-month duration of pain and limitation of range of motion of the shoulder up to 50% as compared to unaffected shoulder, no improvement after conservative treatment involving pharmacotherapy (NSAIDs, steroids drugs in intra-articular injections) and physical therapy, no history of major trauma of shoulder, no signs of rotator cuff tear (as assessed by MRI or ultrasound scan), and occasional sports activity. The exclusion criteria in this study were as follows: history of major trauma of the shoulder, partial or full thickness rotator cuff tear, current or former high-level sport activity, concomitant diabetes mellitus, rheumatoid arthritis, osteoarthritis of the shoulder, and thyroid diseases. All the patients underwent arthroscopic capsular release of the shoulder joint during their stay in hospital.

Following admission to the department, limitation of ROM in the affected and nonaffected shoulder joint was determined by means of a goniometer. Moreover, passive motion range was assessed in the operation room under general anaesthesia, directly before the procedure. Before the surgery, the function of operated shoulder was graded according to ASES (America Shoulder and Elbow Surgeons) score.

**Figure 1:**
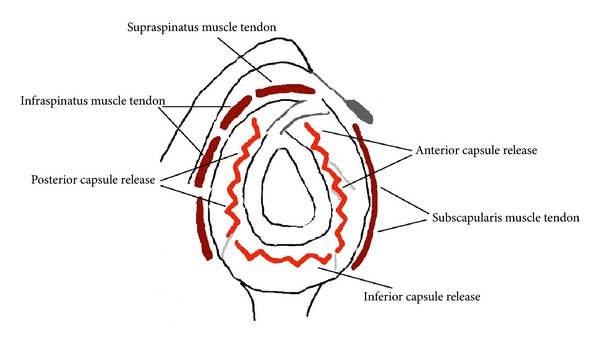
Schema of arthroscopic capsule release of the shoulder.

**Figure 2:**
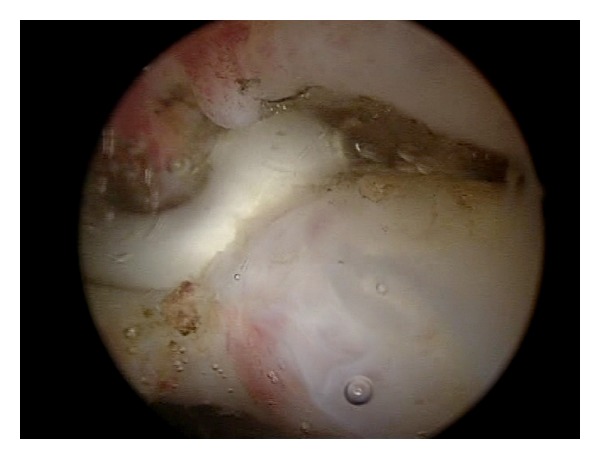
The arthroscopic view of superior capsule release.

**Figure 3:**
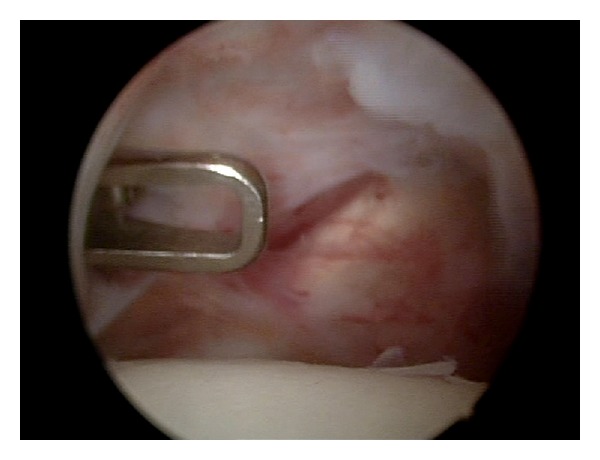
The arthroscopic view of anterior capsule release.

**Figure 4:**
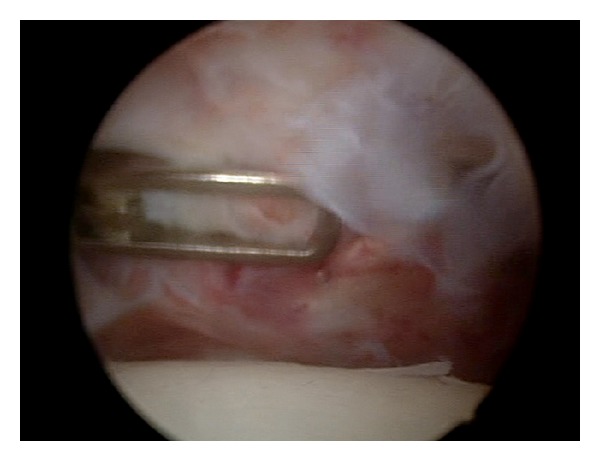
The arthroscopic view of anterior and superior capsule release close to subscapularis muscle tendon.

Arthroscopic capsular release was performed under general endotracheal anaesthesia with patient in the beach-chair position, with trunk flexed at 60° to the lower limbs. All the patients underwent arthroscopic superior, anteroinferior, and posterior capsular release including interval capsule incision [8, 17–21] (Figures 1, 2, and 3). Then gentle manipulation of the shoulder joint was performed. We have always paid attention not to damage subscapularis tendon during anterior capsular release (Figure 4).

**Figure 5:**
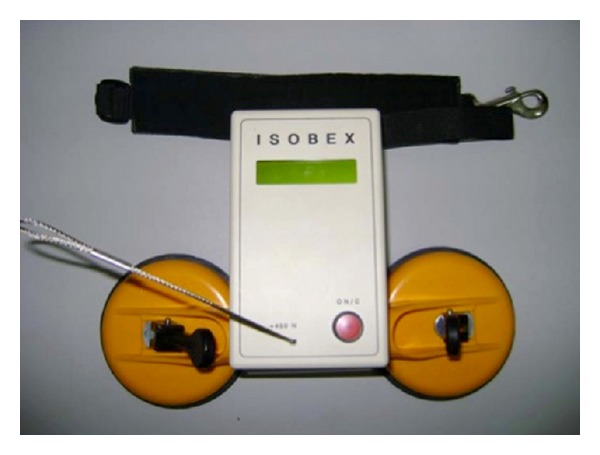
ISOBEX 2.1 dynamometer (CURSOR AG, Bern, Switzerland).

After at least two-year follow-up after the surgery, isometric muscular strength of anterior flexors, abductors, and external and internal rotators of the arm was measured (with arm adducted to the trunk and elbow flexed at 90°). Measurements were performed by means of ISOBEX 2.1 dynamometer (CURSOR AG, Bern, Switzerland) with computer-assisted strength measurement within 5 seconds (Figure 5). The measurements were performed with patients in standing position. Each measurement was performed three times and mean value was calculated. Range of motion of the operated and nonaffected shoulder was determined by means of a goniometer. The function of operated and nonoperated shoulder was also graded according to ASES (America Shoulder and Elbow Surgeons) score.

The study protocol was approved by the local bioethics committee (Approval number RNN/61/07/KB).

Results were processed statistically by means of Statistica PL software. Analysis of results involved Mann-Whitney test, paired difference test (Wilcoxon rank test), and Friedman tests. Nonparametric tests were used for statistical analysis because the data did not follow normal distribution according to Shapiro-Wilk test.

To evaluate the intraobserver and interobserver repeatability of dynamometer measurements, the strength of abduction of both upper limbs was assessed in 10 healthy volunteers twice by the same researcher (R1′ and R1′′) and by the second author (R2). Linear regression analysis was used to calculate the *R* ^2^ value which indicates the level of convergence. The bias was assessed by means of the Bland-Altman plot, which visualizes the percentage difference between 2 measurements (*y*-axis) against their mean (*x*-axis).

## 3. Results

Assessment of shoulder muscular strength was performed by means of ISOBEX dynamometer for four muscle groups: abductors, anterior flexors, and external and internal rotators of the arm. Measurement of isometric muscular strength was performed for nonaffected and operated shoulder after at least two-year follow-up after arthroscopic capsular release of frozen shoulder. No statistically significant differences for arm abductor, flexor, and internal rotators muscular strength between operated and nonaffected shoulders were found (*P* > 0.05) (Table 1). However, difference in measured muscular strength of external rotators between operated and nonaffected shoulders was approximately 8% and proved to be statistically significant (*P* < 0.05) (Table 1).

We did not notice statistically significant differences between dominant and nondominant sides (*P* > 0.05). The differences in muscular strength between dominant and nondominant sides among healthy individuals did not reveal statistical significance (Table 2).

In all the patients, after at least two-year follow-up after the surgery, statistically significant improvement within range of motion in all the planes (flexion, abduction, external rotation, and internal rotation) was obtained (*P* < 0.05), when compared with the period before the surgery. Mobility of the operated shoulder after two-year follow-up within anterior flexion, abduction, and external and internal rotation did not differ significantly as compared to the nonaffected one (Table 1).

The improvement of function of the shoulder after arthroscopic capsular release was also significant (*P* < 0.05), according to America Shoulder and Elbow Surgeons score (ASES, from 25,6 preoperatively to 91,2 postoperatively, 0–100). There were no statistical differences in shoulder function between operated and nonoperated side according to ASES after at least two-year follow-up after the surgery (*P* > 0.05) (Table 1).

The power of statistical tests used in this analysis was assessed to be 80%. The intraobserver and interobserver rates of convergence were *R* ^2^ = 0.993 and *R* ^2^ = 0.995, respectively. Details concerning limits of convergence and bias are presented in Bland-Altman plots. The Bland-Altman plot and *R* ^2^ value confirm that application of the electric dynamometer is a reliable method for evaluating muscular strength throughout the range of parameters measured.

## 4. Discussion

This study involved the assessment of impact of arthroscopic capsular release of frozen shoulder on muscular strength of the operated shoulder and comparison of the results obtained with nonaffected shoulder. Muscular strength of abductors, flexors, external rotators, and internal rotators of the arm was measured. We also tried to determine whether good clinical outcomes according to ASES score correlated with good muscle strength of operated shoulder. In the group of patients with primary frozen shoulder, we did not find statistically significant differences in muscular strength of shoulder, when comparing the operated shoulder with nonaffected one (*P* > 0.05). We also revealed that improvement of function (ASES, *P* < 0.05) after arthroscopic capsular release correlated with good muscular strength of operated shoulder after two-year follow-up. However, a small but statistically significant decrease of muscular strength of external rotators was noted (*P* < 0.05). Unfortunately we could not find out why only in this muscle group did such differences occur. It could result most likely from the atrophy of the muscle group during prolonged shoulder dysfunction before the surgery.

Recent worldwide studies suggest that arthroscopic capsular release is an effective treatment of shoulder contractures in patients that did not respond to prolonged (months) conservative management [8, 17, 22–24]. They all revealed some improvement in the range of motion and function. There are not literature data available concerning the assessment of shoulder muscular strength following arthroscopic surgery for frozen shoulder. The only paper that partially involves the issue is a study by Liem et al. [25]. The authors analysed results of measurement of isometric and isokinetic strengths of external and internal rotators of the arm in patients after arthroscopic capsular release that additionally involved cutting of intra-articular portion of subscapularis tendon. However, the aim of their study was to evaluate the effect of cutting of subscapularis tendon on the muscular strength of rotators as compared to nonoperated shoulder. They did not find statistically significant differences within these parameters between operated and nonaffected shoulder in a group of 22 patients. They also did not notice statistically significant differences between dominant and nondominant sides.

Authors of the relevant literature available agree that some differences in muscular strength of particular shoulder muscle groups dominant and nondominant sides may occur, amounting even to 10% [26, 27]. However, they are not statistically significant. These data are consistent with those obtained from this study. The differences in muscular strength between dominant and nondominant sides were not statistically significant (*P* > 0.05).

Analysis of the measurements of muscular strength in frozen shoulder patients following arthroscopic capsular release suggests that most likely the treatment does not impact muscular strength of the operated shoulder. However, a small number of individuals included in this study may provide a limitation on drawing final conclusions. It also seems important to analyse shoulder muscular strength in such patients in early postoperative period to capture its actual deficit resulting from prolonged preoperative period of shoulder dysfunction and to determine optimal rehabilitation programme.

## 5. Conclusions

The arthroscopic capsular release most likely does not have any impact on the decrease in the muscular strength of the operated shoulder. Improvement of global function of the shoulder (ASES) after arthroscopic capsular release correlates with good muscular strength of the operated shoulder. Undoubtedly, more studies involving higher number of individuals are needed to confirm this hypothesis and conclusions.

## Figures and Tables

**Table 1:** The assessment of range of motion, function, and muscle strength of flexors muscle of the shoulder (FFLX), abductors (ABD), and external (ER) and internal rotators (IR) of the operated and healthy shoulder after minimum two-year follow-up.

	Operated shoulder		Healthy shoulder	*P* value
Number of patients (shoulder)	27			
Dominant/nondominant	15/12			
Mean age (years)	51.6 (24–76)			
F/M	17/10			
Muscular strength (kg)				
FFLX	5.1		5.2	>0.05
ABD	5.0		5.4	>0.05
ER	5.6		6.1	<0.05
IR	6.4		6.7	>0.05
Range of motion (°)	Preop.	Postop.		
FFLX	81.9	166.3	172.1	<0.05
ABD	60.8	147.5	153.4	<0.05
ER	6.1	57.8	64.2	<0.05
IR	Buttock	Th10	Th9	<0.05
Function (ASES, 0–100)	25.6	91.2	93.8	<0.05

**Table 2:** Differences of muscular strength between body sides.

Movement		ABD	FL	ER	IR
Mean strength (SD) [kg]	Dominant	5.2 (2.58)	5.38 (2.8)	5.85 (2.78)	6.26 (2.88)
	Nondominant	5.52 (2.82)	5.54 (2.97)	5.45 (2.22)	6.29 (2.96)
*P* value		0.35	0.60	0.22	0.89
